# SCT-Diff: Seamless Contextual Tracking via Diffusion Trajectory

**DOI:** 10.3390/jimaging12010038

**Published:** 2026-01-09

**Authors:** Guohao Nie, Xingmei Wang, Debin Zhang, He Wang

**Affiliations:** College of Computer Science and Technology, Harbin Engineering University, 145 Nantong Street, Harbin 150000, China; nieguohao@hrbeu.edu.cn (G.N.); zhangdebin@hrbeu.edu.cn (D.Z.); b424060006@hrbeu.edu.cn (H.W.)

**Keywords:** visual object tracking, diffusion model, trajectory fitting, temporal relation modeling

## Abstract

Existing detection-based trackers exploit temporal contexts by updating appearance models or modeling target motion. However, the sequential one-shot integration of temporal priors risks amplifying error accumulation, as frame-level template matching restricts comprehensive spatiotemporal analysis. To address this, we propose SCT-Diff, a video-level framework that holistically estimates target trajectories. Specifically, SCT-Diff processes video clips globally via a diffusion model to incorporate bidirectional spatiotemporal awareness, where reverse diffusion steps progressively refine noisy trajectory proposals into optimal predictions. Crucially, SCT-Diff enables iterative correction of historical trajectory hypotheses by observing future contexts within a sliding time window. This closed-loop feedback from future frames preserves temporal consistency and breaks the error propagation chain under complex appearance variations. For joint modeling of appearance and motion dynamics, we formulate trajectories as unified discrete token sequences. The designed Mamba-based expert decoder bridges visual features with language-formulated trajectories, enabling lightweight yet coherent sequence modeling. Extensive experiments demonstrate SCT-Diff’s superior efficiency and performance, achieving 75.4% AO on GOT-10k while maintaining real-time computational efficiency.

## 1. Introduction

Visual object tracking (VOT) aims to precisely estimate the location of a target object across consecutive frames in a video sequence. Modern trackers frequently utilize a detection-based framework, predicting the target’s state in each frame via a localized search window approach [1,2]. As both the target and its environment evolve dynamically, depending exclusively on static appearance cues—such as the initial template—to localize the target in subsequent frames proves insufficient for addressing dynamic and complex scenarios [3,4,5,6,7,8].

In light of the aforementioned challenges, numerous models have explored spatiotemporal contexts to address appearance variations, including template update strategies [9,10], integrated historical appearance representations [11,12,13,14], and motion trajectory modeling [15,16,17,18,19,20,21]. However, despite these efforts, existing methods remain constrained by frame-by-frame template matching principles and restrict object state prediction to the current search frame [7,22]. The one-shot integration of temporal priors [4,5,23] introduces inherent limitations: (1) Insufficient global spatiotemporal integration. Current methods primarily focus on merging past tracking results but fail to holistically model the global dynamics of object motion and appearance over video sequences. This prevents validation and refinement of predictions using future frames, thereby limiting robustness to significant appearance changes and disturbances. (2) Lack of temporal propagation coherence. Prediction errors or noise can propagate and amplify during online updates, ultimately resulting in tracking failure. Furthermore, the inability to roughly estimate targets beyond the immediate search window hinders effective exploitation of continuous trajectory patterns.

**Figure 1:**
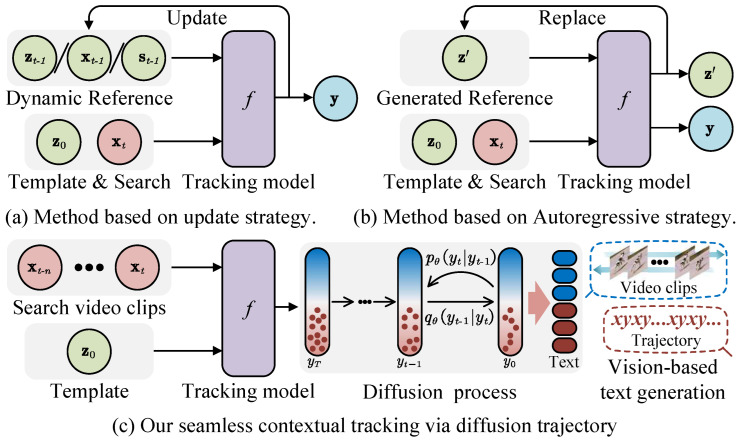
Compared to detection-based trackers that employ spatiotemporal information. (Green: past frames & references; Red: current search frame; Purple: tracking model; Blue: tracking results; Darker red: textual features; Darker blue: visual features.) (**a**) The dynamic references are updated based on tracking results. (**b**) The temporal autoregressive strategy generates a reference feature to guide tracking in the subsequent frame. (**c**) Our method takes a video clip as input and jointly infers the entire trajectory using the diffusion model.

To address these issues, we introduce a tracking framework within a "seamless context", defined as continuous observation without temporal sampling gaps. The model simultaneously estimates object trajectories across a video clip within a temporal window, as illustrated in Figure 1. By maintaining uninterrupted target and background representations, the model explicitly mimics bidirectional continuity in object spatiotemporal variations. This enables the estimation of current target states from historical trajectories and the refinement of prior predictions using subsequent observations. Such comprehensive spatiotemporal analysis intuitively surpasses conventional frame-by-frame template matching, which typically references discrete temporal information. To this end, we generalize traditional frame-level tracking to video-level trajectory inference. A denoising diffusion process [24,25,26] is constructed to progressively refine random trajectory hypotheses through multiple diffusion steps. Unlike methods relying on one-shot temporal prior integration, seamless context at the video clip improves the perception of temporal changes.

In this work, we propose a novel tracking framework, SCT-Diff. SCT-Diff employs a diffusion model to achieve video-level tracking within temporal windows, eliminating the need for cumbersome or temporal-specific components. The encoder-decoder architecture is utilized to holistically address global appearance variations and motion dynamics. Inspired by language modeling paradigms, the framework represents target trajectories in videos as discrete token sequences [17]. The encoder extracts frame-level features that capture object-aware information. To fully leverage spatiotemporal relationships across frames, we propose a Mamba-based expert decoder network. Each decoder block integrates a lightweight vision expert layer to incorporate coherent video feature representations. Concurrently, several language expert layers progressively refine trajectory estimation during the denoising diffusion process, formulating VOT as a vision-conditioned diffusion text generation. During training, the decoder learns to predict denoised trajectories from Gaussian-noised inputs, while inference reverses this diffusion process. Vision and trajectory features serve as mutual prompts, facilitating bidirectional propagation of appearance and motion cues within temporal windows. This global spatiotemporal analysis allows for the correction of target predictions at any time point, enhancing tracking consistency. Consequently, the framework mitigates error propagation risks across frames.

Extensive experiments on large-scale VOT benchmarks show that our proposed SCT-Diff outperforms recent state-of-the-art trackers. For instance, under fair conditions, SCT-Diff-B256 obtains a 75.4% AO score on the GOT-10k dataset, surpassing OSTrack [23] by 4.4% and ARTrack [15] by 1.9%.

In summary, the contributions of this work are as follows:

- We propose SCT-Diff, a video-level diffusion tracking framework designed to holistically reconstruct the tracking trajectory. This enables bidirectional spatiotemporal perception, overcoming the limitations of static template matching and one-shot temporal priors integration.
- We introduce a novel decoder architecture incorporating Mamba-based lightweight vision-language experts, seamlessly bridging global context aggregation for motion and appearance dynamics.
- A non-causal interaction mechanism exploits future situations to facilitate self-correction of trajectory hypotheses. This exploits temporal propagation consistency to mitigate update risk. Extensive results from the large-scale VOT benchmark demonstrate the effectiveness of the proposed method.

This paper is structured as follows: Section 2 reviews relevant prior work in the field. Section 3 introduces the proposed methodology and object-tracking framework. In Section 4, we conduct a detailed performance evaluation and effectiveness analysis of our approach. Finally, Section 5 concludes the paper and outlines potential directions for future research.

## 2. Related Work

As the first step, this paper reviews visual object tracking methods relevant to this work, including temporal relation modeling approaches, and provides a brief introduction to the Diffusion model.

### 2.1. Visual Object Tracking

Trackers utilizing the Siamese paradigm [5,27] perform similarity matching between template and search regions to achieve target localization. These systems sequentially localize objects by cropping search regions based on bounding box predictions from previous frames. The integration of relation modeling [4,23,28], prediction frameworks [29,30,31], and vision transformer models [6,7,9,32] has significantly advanced modern tracking systems. Under the one-shot detection framework [5], models struggle to adapt to changes in the target and environment over time, making them easily drift towards distractors. To address these issues, various dynamic optimization modules are employed, such as discriminative correlation filters [12,33,34,35], template update mechanisms [7,13,36], model fine-tuning [37,38], and target memory [39,40,41,42,43]. Although the single forward pass evaluation scheme is effective, it impedes the continuous propagation of spatiotemporal information in both forward and backward directions. The disturbance prediction of the previous time step is difficult to correct based on the current continuous target movement. Therefore, we reformulate the tracking problem as the iterative refinement of a continuous trajectory, explicitly modeling bidirectional temporal dependencies.

### 2.2. Temporal Relation Modeling

Numerous mainstream studies investigate temporal information in tracking to explore change patterns of target states, generally categorized into appearance [7,9,44,45,46] and motion variations [6,18,19,47]. The most prevalent technique involves updating target templates to accommodate appearance changes. Moreover, several approaches further integrate historical appearance for comprehensive temporal information utilization, such as UpdateNet [13], THOR [48] and STMTrack [49]. However, these methods are forced to employ artificially designed complex rules to mitigate update-related risks [40,41,50]. To preserve extended historical frames, the learned feature representations are introduced to encapsulate prior appearance [11,14]. Beyond appearance adaptation, a few studies [6,47] focus on learning a feature to describe the target’s previous state or motion information. Moreover, trajectory prediction [14,16,17,18,19,20,51] enhances traditional continuous motion assumptions by capturing kinematic trends. Models can implement soft attention mechanisms for potential target localization based on short-term historical trajectories [19]. The autoregressive trackers predict coordinates through historical sequences and current search features [14,17]. However, the historical visual features remain underutilized in this process. To address this limitation, we propose seamless reasoning over continuous target trajectories within video segments, thereby holistically integrating appearance variation and motion trends.

### 2.3. Diffusion Model

Diffusion models have demonstrated remarkable success in computer vision [52,53,54,55,56,57] and audio processing [58,59,60,61,62], where data inherently exists in a continuous form. As generative models, they learn the data generation distribution by simulating the diffusion of data through Gaussian noise. While diffusion models are not inherently designed for discrete data, recent approaches address discrete language tasks by mapping tokens to continuous embedding spaces [63,64,65,66,67]. Notably, diffusion frameworks have also shown promise in discriminative tasks [68,69], including semantic segmentation through mask prediction [70,71,72,73,74]. For instance, DiffusionDet [75] reformulates object detection as a denoising process that refines noisy bounding boxes into target boxes, while DiffTrack [76] extends this paradigm to visual object tracking via point set diffusion. However, modeling detection-based tasks in a continuous space introduces significant complexity compared to traditional tracking methods, which update a limited number of noisy boxes. This paper aims to enhance and balance the performance of diffusion-based tracking models by leveraging discrete language representations.

## 3. Methodologies

In this section, we first outline the preparatory steps required for applying continuous diffusion models to trajectory generation. Subsequently, we introduce the proposed SCT-Diff model architecture. Finally, we discuss the training and inference procedures.

### 3.1. Preliminaries

#### 3.1.1. Spatiotemporal Tracking Framework

Given the initial template *z* and the search image *x*, the objective of visual tracking is to determine the current state of the target. The tracking approach typically involves learning a model *f* to estimate the target’s position and scale.

(1) $$b_{\tau }=f\left(z,x_{\tau },r_{:\tau -1}\right),$$

The reference information, $r_{:\tau -1}$, is updated during tracking and includes dynamic templates, historical search areas, and target trajectories. The bounding box *b* is described by a sequence of coordinates $\left[x_{\min},y_{\min},x_{\max},y_{\max}\right]$.

#### 3.1.2. Diffusion Model Framework

Diffusion models represent data $i_{0}\in R^{d}$ as a Markov chain $i_{T},\ldots ,i_{0}$, where each latent variable $i_{t}$ is in $R^{d}$, and $i_{T}$ is Gaussian. Given the initial state $p\left(i_{t}\right)\approx N\left(0,I\right)$, the diffusion model progressively denoises the sequence $i_{T:1}$ to approximate samples from the target data distribution, parameterized as $p\left(i_{t-1}|i_{t}\right)=N\left(i_{t-1};f_{\theta }\left(i_{t},t\right),\Sigma_{\theta }\left(i_{t},t\right)\right)$. To train the diffusion model, a forward process obtains intermediate latent variables by adding noise to $i_{0}$, represented as $q\left(i_{t}|i_{t-1}\right)=N\left(i_{t};\sqrt{1-\beta_{t}}i_{t-1},\beta_{t}I\right)$. The hyperparameter $\beta_{t}$ is the amount of noise added in diffusion step *t*. The training objective is to generate noisy data according to the predefined forward process *q* and train the model to reverse this process and reconstruct the data. The reverse process is supervised by minimizing the $\ell_{2}$ loss:

(2) $$\ell_{train}=\frac{1}{2}{∥f_{\theta }\left(i_{t},t\right)-i_{0}∥}^{2}.$$

During the inference process, the model progressively reconstructs data samples $i_{0}$ from noise using iterative methods.

### 3.2. SCT-Diff Framework

**Figure 2:**
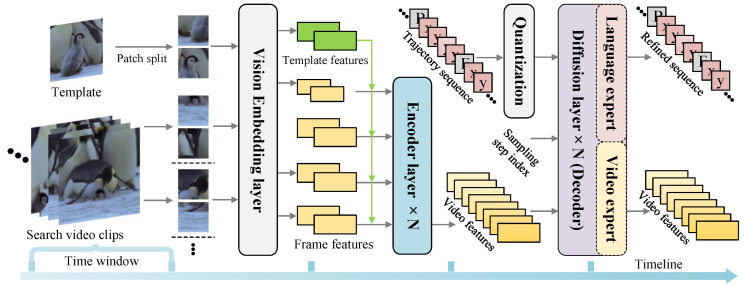
Architectureof the proposed SCT-Diff. The encoder extracts target-aware features and passes the search features to the decoder. The decoder, composed of a series of diffusion layers, refines the trajectory to estimate target states.

We depict the target trajectory using a sequence of discrete tokens and then introduce the model architecture. The overall framework is illustrated in Figure 2, which comprises a transformer-based encoder and a diffusion-based decoder.

#### 3.2.1. Trajectory Coordinate Tokenization

SCT-Diff operates within a time window Δτ to estimate a series of object states $b_{\tau -\Delta \tau :\tau }=\left[b_{\tau -\Delta \tau },\cdots ,b_{\tau }\right]$ corresponding to a video segment. In contrast, previous detection-based methods conduct prediction on individual frames. We apply tokenization [15,17] to represent each coordinate in $b_{\tau -\Delta \tau :\tau }$ as an integer between the vocabulary $\left[1,n_{bins}\right]$, thereby avoiding the large number of parameters brought about by describing continuous coordinates. To accommodate fast-moving objects that may exceed image boundaries, we first expand the coordinate representation range for both the search region and its normalized target bounding box to $\left[-0.5,1.5\right]\times$ the search area. We then linearly map each coordinate value to an integer in $\left[1,n_{bins}\right]$, clipping out-of-range values at the boundaries. All coordinates share a unified vocabulary of $n_{bins}$ discrete tokens, with each token corresponding to a learnable embedding vector. We represent per-frame target locations using the corner format, which encodes each frame’s trajectory into exactly 4 coordinate tokens. For a video clip spanning Δτ frames, the full trajectory sequence length becomes 4×Δτ tokens. To enable efficient batch training, we fix Δτ to a constant value (Δτ=6 in our experiments), yielding a uniform sequence length of 24 tokens per sample. Tracking trajectories become texts composed of sequences of discrete words. Considering speed, tracking is usually performed within a local window. To be consistent with the previous framework, we generate a complete search video segment $x_{\tau -\Delta \tau :\tau }$ by extracting the window from each frame of the video segment based on a unified position reference. The tracking trajectory is subsequently mapped to the same coordinate system. This step can be omitted if the search range encompasses the entire frame.

#### 3.2.2. Diffusion Models for Trajectory Generation

Within the interval Δτ, trajectory prediction is formulated as controllable text generation where $b_{\tau -\Delta \tau :\tau }$ is sampled from the conditional distribution $p\left(b_{\tau -\Delta \tau :\tau }|x_{\tau -\Delta \tau :\tau },z\right)$. The canonical approach in language modeling predicts the next token based on the generated sequence in an autoregressive manner. Equation (1) follows an approximately autoregressive process over time. The estimated target state in the current frame is influenced by adjacent preceding target states and also affects subsequent frames. However, this strict sequential inference always assumes that the previous tracking results are reliable. This may amplify the risk introduced by updates and make it difficult to correct prior erroneous predictions based on bidirectional temporal continuity.

To this end, we model the visual tracking task as a diffusion process to integrate global spatiotemporal information. The sequence of discrete words $b_{\tau -\Delta \tau :\tau }$ represents a series of target states within continuous spacetime. Applying the continuous diffusion model necessitates both an embedding step and a rounding step between $b_{\tau -\Delta \tau :\tau }$ and $i_{0}$. The sequence $w_{\tau -\Delta \tau :\tau }$ is defined as $w_{\tau -\Delta \tau :\tau }=\left[\mathrm{Emb}\left(b_{\tau -\Delta \tau }\right),\ldots ,\mathrm{Emb}\left(b_{\tau }\right)\right]$. In the reverse process, a softmax-based trainable rounding step, denoted as $p\left(w|i_{0}\right)$, is incorporated. Consequently, Equation (2) is adjusted accordingly:

(3) $$\ell_{train}=\frac{1}{2}{∥w-i_{0}∥}^{2}-\log p_{\theta }\left(w|i_{0}\right).$$

The goal of the noise-to-trajectory paradigm is to learn a tracking model *f* that can progressively refine trajectory estimates over a total of *T* steps:

(4) $$w_{N}^{T}\overset{f}{\to }w_{N}^{T-\Delta T}\overset{f}{\to }\cdots \overset{f}{\to }w_{N}^{0},$$

where diffusion step T→0 depicts the target estimation changes from an absolute random state to the highest certainty. ΔT is the time interval of the diffusion step. Thus, the tracking process based on the diffusion model $f_{\theta }$ can be formulated as

(5) $$w_{N}^{t}=f_{\theta }\left(z,x_{\tau -\Delta \tau :\tau },t,w_{N}^{t-\Delta t}\right).$$

The model $f_{\theta }$ refines the current estimate $w_{N}^{t}$ by utilizing diffusion step index *t* and incorporating the estimation from the prior step.

#### 3.2.3. Encoder

SCT-Diff utilizes a general Vision Transformer (ViT) [32] image encoder in accordance with the OSTrack [23] principles. To enhance encoding efficiency, individual frames within the interval Δτ are encoded separately, rather than processing video segments. Initially, the template and search images are segmented into patches. These patches are then flattened and projected to create a series of token embeddings. Positional embeddings are added to both template and search tokens. The concatenated tokens are subsequently fed into the ViT backbone to jointly extract visual features and learn feature-level correspondences.

#### 3.2.4. Decoder

**Figure 3:**
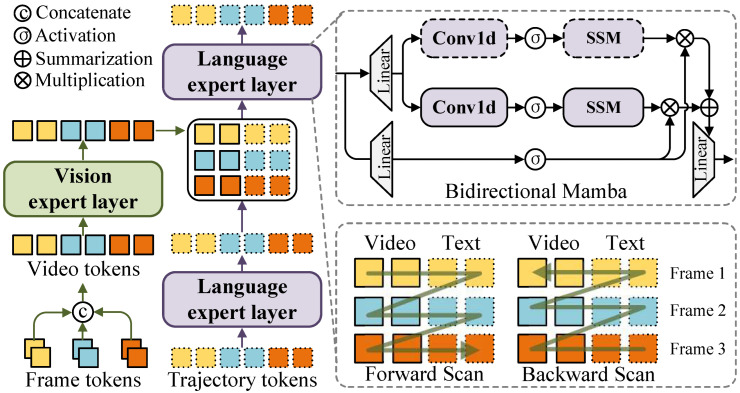
Structure of the diffusion block. It consists of Mamba-based vision and language experts to model continuous changes in appearance and motion, respectively.

The decoder of SCT-Diff is a stack of diffusion blocks. As illustrated in Figure 3, each diffusion block comprises vision expert and language expert layers. The diffusion block processes the trajectory embedding tokens $w_{\tau -\Delta \tau :\tau }=\left[w_{\tau -\Delta \tau },\cdots ,w_{\tau }\right]$ and frame features $F_{\tau -\Delta \tau :\tau }=\left[F_{\tau -\Delta \tau },\cdots ,F_{\tau }\right]$ from the previous diffusion step t−Δt. Initially, a vision expert layer manages the continuous appearance evolution of the target and background. The frame features are linked chronologically to create video features. Accurately locating objects in a video requires a model that can handle long videos with high resolution. We utilize a 3-D convolutional layer to project $F_{\tau -\Delta \tau :\tau }\in R^{L\times C}$, thereby generating spatiotemporal tokens of uniform size, where L=τ×H/16×W/16.

$F_{\tau -\Delta \tau :\tau }$ and $w_{\tau -\Delta \tau :\tau }$ are assigned 2-dimensional and 1-dimensionalposition embeddings, respectively. The flattened visual and trajectory features are combined with identical temporal embeddings. Considering real-time video trajectory inference, we then employ a state-space model (SSM) with linear complexity to model global spatiotemporal relationships.

(6) $$F_{t-\Delta t}:=Mamba\left(Conv3d\left(F_{t-\Delta t}\right)\right).$$

SSM is developed for continuous systems that map a 1D function or sequence $x\left(t\right)\in R^{L}\to y\left(t\right)\in R^{L}$ through a hidden state $h\left(t\right)\in R^{N}$. Formally, SSMs employ the following ordinary differential equation (ODE) to model the input data:

(7) $$\begin{array}{cc} h^{\prime }\left(t\right) & =Ah\left(t\right)+Bx\left(t\right),\\ y\left(t\right) & =Ch\left(t\right), \end{array}$$

where $A\in R^{N\times N}$ represents the system’s evolution matrix, and $B\in R^{N\times 1}$, $C\in R^{N\times 1}$ are the projection matrices. This continuous ODE is approximated through discretization in modern SSMs, such as Mamba [77]. Mamba introduces a time scale parameter and a selective scanning mechanism to achieve efficient long-sequence modeling capabilities. For more details, please refer to [77]. Subsequently, the language expert layer is used to process the motion information of the target trajectory. For convenience, it adopts a similar Mamba architecture:

(8) $$w_{t-\Delta t}:=Mamba\left(w_{t-\Delta t}\right),$$

Finally, vision and trajectory information are integrated to predict the refined trajectory states through an additional language expert. In a seamless spatiotemporal context, the appearance and motion of the target are continuous over time. Therefore, we employ a bidirectional Mamba (B-Mamba) model to extend spatiotemporal perception and processing capabilities simultaneously. Therefore, a bidirectional 3D bimodal scan is set up for spatiotemporal input, as shown in Figure 3. We organize spatial annotations in a vision-language sequence, stacking them frame by frame to maintain the correct timeline. Furthermore, the experiments, as shown in ablation study, demonstrate that this sequential integration scanning method is the most effective. Notably, the trajectory and video prompts enhance each other reciprocally. They are jointly passed to the next diffusion block, but only the trajectory token embeddings are diffused.

### 3.3. Training

The objective of the diffusion tracking model $f_{\theta }$ is to predict the ground truth of target states $b_{0}$ from random noisy conditions $b_{t}$, represented as $q\left(b_{0}|b_{t}\right)$. The noise $N_{e}$ is combined with the ground truth $b_{0}$ to generate a training input $w_{N}^{t}$. The noise scale for each time step *t* is controlled by a pre-defined monotonically decreasing schedule proposed in [78,79].

#### 3.3.1. Training Loss

The diffusion decoder takes noisy trajectories and video features as input. It employs the softmax cross-entropy loss function to maximize the log-likelihood of the token sequence. However, the classification loss overlooks the physical properties of the tokens, such as the spatial relation of coordinates. To address this, we decompose the trajectory sequence into independent bounding boxes and calculate their SIoU with the ground truth values, thereby incorporating tracking-related task knowledge. The overall loss function of SCT-Diff is as follows:

(9) $$L=L_{ce}+\lambda L_{iou},$$

where $L_{ce}$ and $L_{iou}$ are the cross-entropy loss and SIoU loss, respectively, and λ is a weight to balance the two loss terms.

#### 3.3.2. Two-Stage Training

Existing research mostly predicts the targets of each frame independently and then concatenates these predictions into target trajectories. In contrast, SCT-Diff decodes target trajectories directly from short video clips. Under the same training sample pairs, this requires more memory and computation, leading to longer training times. From a visual language perspective, we propose a two-stage training strategy. The first stage learns robust appearance representations similar to object detection, and the second stage fine-tunes the temporal dynamics on this solid foundation, achieving a 1.6-point AUC gain (See the ablation of training strategy). This curriculum-like approach prevents the diffusion process from being overwhelmed by temporal noise early on. If Δτ=0, our method aligns with other per-frame trained trackers. Each vector of w is padded with [Begin] and [End] markers to inform the model of the estimated range of the sequence at the current moment. These markers pass through the embedding layer and form a fixed-size tensor of [Batch-size, Seq-len, Embedding-dim], which can be applied to continuous diffusion. Subsequently, the model is trained using the same approach as OSTrack [23] and STARK [7], resulting in SCT-Diff(0). SCT-Diff(0) predicts coordinates between the [Begin] and [End] markers. During the second stage, we extend the time span to Δτ>0 and fine-tune the SCT-Diff(0) model. [Begin] and [End] tokens identify each frame’s coordinate sequence range. Under this configuration with Δτ=6, the total input sequence length is 36 tokens. After several rounds of fine-tuning, the model incorporates spatiotemporal information into its predictions.

### 3.4. Inference

The inference procedure of the tracking model is a denoising sampling process from noise to target. The tracking model samples coordinates from random noise and iteratively refines the trajectory. When generating sequences, a similar rounding function is employed to map the denoised text embeddings to the nearest discrete tokens. These denoised text embeddings are passed through a linear layer to obtain the logits, after which a softmax function is used to determine the probability of each token. The token with the highest probability is selected as the predicted token in the trajectory. The continuous coordinates form the trajectory of the target within a small segment of spatiotemporal. We divide the long video into smaller segments to gradually generate trajectories.

#### Trajectory Refinement

Traditional methods predict the target in a single forward evaluation. In contrast, our approach corrects previous trajectory by adjusting the stride of the sliding time window and incorporating future visual information. At each evaluation step, the previous target trajectory is truncated by the stride length. By sliding the time window, we fill in the latest frames and add noise to restore the video and trajectory sequences. When the coordinate classification score exceeds the previous scores, the predicted coordinates are corrected using estimates from future frames. Although SCT-Diff operates on videos, the multi-frame prediction does not incur unacceptable computational overhead. The detailed decoding algorithm is presented in Algorithm 1.
**Algorithm 1 **Inferencealgorithm (decoder only)**Require:** 
trained $\mathrm{Decoder}\left(\cdot \right)$, search window features $F_{\tau -\Delta \tau :\tau }$, and previous trajectory tokens $w_{\tau -\Delta \tau :\tau -\Delta \tau /2}$.**Ensure:** 
corrected trajectory $b_{\tau -\Delta \tau :\tau -\Delta \tau /2}$, new predicted trajectory tokens $w_{\tau -\Delta \tau /2:\tau }$.  1:**for** each time window Δτ in tracking sequence **do**  2:    Initialize trajectory estimation $w_{\tau -\Delta \tau /2:\tau }$ from a random distribution.  3:    Concatenate complete trajectories $w_{\tau -\Delta \tau :\tau }$  4:    Obtain diffusion step index *t* for current evaluation.  5:    Predict results from all *L* diffusion layers. ${\left\{w_{\tau -\Delta \tau :\tau },F_{\tau -\Delta \tau :\tau }\right\}}_{l=1}^{L}\leftarrow {\mathrm{Decoder}}^{L}\left(t,w_{\tau -\Delta \tau :\tau },F_{\tau -\Delta \tau :\tau }\right)$  6:    Replace previous trajectory with current estimates. ${\left\{b_{\tau -\Delta \tau :\tau -\Delta \tau /2}\right\}}^{\left(1\right)}\leftarrow {\left\{b_{\tau -\Delta \tau :\tau -\Delta \tau /2}\right\}}^{\left(2\right)}$  7:    **if** update **then**  8:        Replace dynamic templates from frames $\left\{x_{\Delta \tau /2-\Delta \tau }\right\}$.  9:    **end if**10:**end for**11:**return** $w_{\tau -\Delta \tau /2}$; $b_{\tau -\Delta \tau :\tau -\Delta \tau /2}$.

## 4. Experiments

In this section, we first describe the implementation details of our method. We then present extensive ablation studies and compare our method with advanced trackers. Additionally, we provide qualitative results to showcase the proposed method’s effectiveness.

### 4.1. Implementation Details

SCT-Diff is trained on two NVIDIA GeForce RTX 3090 GPUs utilizing Python 3.8 and PyTorch 1.11.0. A single GPU 3090 is used in the test platform. The training dataset comprises the training splits from COCO [80], LaSOT [81], GOT-10k [82], and TrackingNet [83]. We evaluated our method using four widely-used large datasets: LaSOT [81], TNL2K [84], GOT-10k [82], and TrackingNet [83]. There are three challenging smaller test sets, including OTB-100 [85], NFS [86], and TC-128 [87].

The model input consists of a short video clip and template group. For convenience, the video clip comprises a series of search windows, which are cropped based on the average position of the target in the preceding trajectory. Each search window is set to 4 times the size of the initial target and scaled to 256×256. The video clip has a length of 6 frames. The template is twice the size of the initial target and scaled to 128×128 pixels. In addition to the initial template, a dynamic template is used to record the latest target changes, selecting the target state with the highest classification score in the previous trajectory. ViT-Base [32] serves as the encoder structure in SCT-Diff. The output of the final layer is retained to construct video features, which are then fed into the decoder. Random coordinates are generated through quantization operations to create the corresponding text sequences. The decoder consists of 6 diffusion blocks that interact with the trajectory and video features. The balancing weight λ in Equation (9) is set to a fixed value of 2, following the common practice in the recent tracking literature [15]. No extensive hyperparameter tuning was performed to keep the training protocol simple and generalizable.

As shown by frame-level trackers [15,17], quantization precision positively impacts performance: larger $n_{\mathrm{bins}}$ reduces quantization error and improves localization accuracy, but the gains quickly saturate. Meanwhile, a linearly growing vocabulary proportionally increases the embedding layer parameters and decoder classification overhead. Since our core contribution is leveraging diffusion models for temporally coherent trajectory generation rather than tokenization strategy itself, we empirically select $n_{\mathrm{bins}}=800$ [15] as the optimal balance between accuracy and efficiency. We adopt the cosine noise scheduling [79], which defines $\beta_{t}\in \left[0,0.999\right]$ for T=1000 timesteps. During training, ground-truth boxes are converted into trajectory tokens and noised with standard Gaussian ϵ∼N(0,I) via the forward diffusion process: $i_{t}=\sqrt{{\bar{\alpha }}_{t}}\cdot i_{0}+\sqrt{1-{\bar{\alpha }}_{t}}\cdot \epsilon ,$ where ${\bar{\alpha }}_{t}=\prod_{i=1}^{t}\left(1-\beta_{i}\right)$ and *t* is uniformly sampled from [1,1000]. For inference, we employ the DDIM sampler [88] with a single sampling step for efficient decoding.

The model training comprises two stages. In the first stage, referred to as single-frame training, the model undergoes 240 epochs of training, with 60,000 matching pairs processed per epoch. After 200 epochs, the learning rate is decreased by a factor of ten. The AdamW [89] optimizer is employed with an initial learning rate of $8\times 10^{-5}$ and a batch size of 48. In the second stage, the COCO dataset is excluded. We then conduct an additional 60 epochs of training on randomly sampled video segments from three video datasets, with 35,000 sample pairs processed per epoch.

### 4.2. Overall Performance

We evaluated the performance of the proposed SCT-Diff tracker on seven popular benchmark datasets and compared it with advanced trackers. The evaluation included three smaller datasets (OTB-100 [85], NFS [86], and TC-128 [87]) containing 100, 100, and 128 short-term video sequences respectively, covering diverse scenarios. Success rate and precision are adopted as evaluation metrics for testing and ranking. For larger-scale experiments, four major datasets (LaSOT [81], TNL2K [84], GOT-10k [82], and TrackingNet [83]) are employed, comprising 280, 700, 180, and 511 test sequences respectively. Three metrics are used to assess the tracker’s performance: Area Under the Curve (AUC), precision (P), and normalized precision (P-Norm). In GOT-10k, the average overlap (AO) and success rate (SR) reported by the official evaluation service served as performance indicators.

#### 4.2.1. GOT-10k

GOT-10k [82] is a large-scale benchmark with over 10k frames and non-overlapping classes in training and testing to evaluate generalization. According to the official protocol, we have trained our SCT-Diff only on the GOT-10k training split. Performance metrics include average overlap (AO) and success rate (SR). The methods involved in the comparison include MDNet [90], ATOM [35], SiamRPN++ [27], DiMP [33], TrDiMP [46], TransT [4], STARK [7], AiATrack [91], SwinTrack-T [6], MixFormer-22k [9], OSTrack [23], GRM [28], EVPTrack [92], ARTrack [15], SeqTrack-B [17], DiffusionTrack [76] and MIMTrack. As shown in Table 1, SCT-Diff achieves scores of 75.4%, 86.7%, and 73.3% for AO, SR_0.5_, and SR_0.75_, respectively. The reported tracking speed during testing is 63.5 FPS. ARTrack and SeqTrack share a trajectory prediction framework similar to our SCT-Diff. In SeqTrack, the decoder integrates visual templates with historical target motion trajectories. ARTrack further incorporates temporal autoregressive training based on target trajectories. However, these methods fail to leverage continuous visual information. In contrast, SCT-Diff performs trajectory prediction using consecutive video clips rather than individual frames, yielding AO improvements of 1.9% and 0.7%. Compared to DiffusionTrack, which employs a diffusion-based framework, our method exhibits a 1.3% gain in SR_0.75_. This enhancement primarily arises from SCT-Diff’s refinement of tracking predictions through global spatiotemporal information. As DiffusionTrack adopts an RPN-like spatial sampling and diffusion strategy, its tracking speed on the same hardware is approximately 35 FPS, which is 45% slower than SCT-Diff. These results demonstrate SCT-Diff’s effective balance between speed and precision.

#### 4.2.2. LaSOT

**Figure 4:**
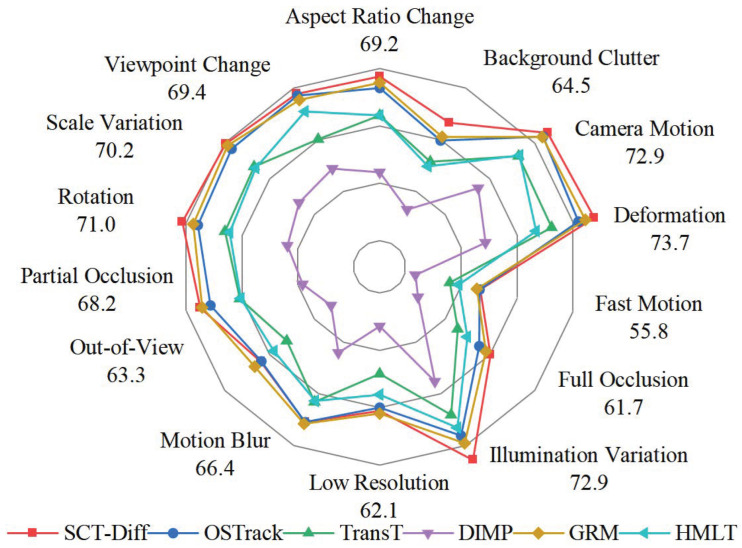
Results for the challenge attributes of the LaSOT dataset.

The comprehensive long-term tracking dataset, LaSOT [81], encompasses 280 test video sequences. As shown in Table 1, SCT-Diff achieves state-of-the-art AUC score (71.1%) and precision scores (77.5%), outperforming SOTA trackers DiffusionTrack and EVPTrack by 0.3%/0.7% in AUC metrics. Under fair 256-resolution conditions, SCT-Diff also demonstrates superior AUC performance compared to OSTrack and MixFormer, with gains of 2% and 1.9% respectively. Without sophisticated modifications, SCT-Diff surpasses both MIMTrack and AiATrack on the LaSOT benchmark. The latter two explore template-free temporal context modeling through image generation and discriminative model paradigms, respectively. Figure 4 visualizes the performance of the proposed method across 14 distinct challenge attributes on the LaSOT dataset. As can be observed, SCT-Diff maintains stable performance under most challenging conditions. By observing the continuous evolution of the target, our method demonstrates advantages in handling continuous variations such as illumination changes and rotation. This also affords certain adaptability to partial occlusion. However, due to constraints in the temporal window range, the superiority becomes less pronounced for longer-term occlusions. These results substantiate the effectiveness of our bidirectional propagation mechanism for target-specific feature representation and motion correlation across temporal dimensions.

#### 4.2.3. TrackingNet

TrackingNet [83] encompasses 511 video sequences depicting various scenes. Table 1 presents the comparative results, where SCT-Diff achieves an AUC score of 84.0%, a P-Norm score of 88.8%, and a P-score of 83.4%. Our method surpasses most Siamese tracking approaches (e.g., TrDiMP, TransT, and STARK). Compared to DiMP and TrDiMP, SCT-Diff exhibits AUC improvements of 10% and 5.6%, respectively. This highlights its superiority in leveraging continuous spatiotemporal information over pure spatial relation modeling. Notably, compared to the multi-template method STARK, the bidirectional mutual validation of temporal information contributes to a 2% AUC gain. SCT-Diff performs comparably to the sequence generation tracker ARTrack but significantly outperforms the similar framework SeqTrack (with a 1.2% precision improvement). This discrepancy may stem from ARTrack’s use of comprehensive temporal autoregressive training, and the smaller and smoother temporal variation of TrackingNet narrows the performance gap between trackers. Overall, SCT-Diff still demonstrates advantageous performance.

#### 4.2.4. TNL2K

TNL2K [84], a recently released large-scale dataset, comprises 700 challenging video sequences. As shown in Table 1, our SCT-Diff significantly outperforms all other trackers, achieving state-of-the-art performance with 58.5% AUC - surpassing ARTrack by 1% margin. This demonstrates substantial improvements in tracking robustness and accuracy across diverse challenging scenarios.

#### 4.2.5. OTB-100, NFS, and TC-128

**Figure 5:**
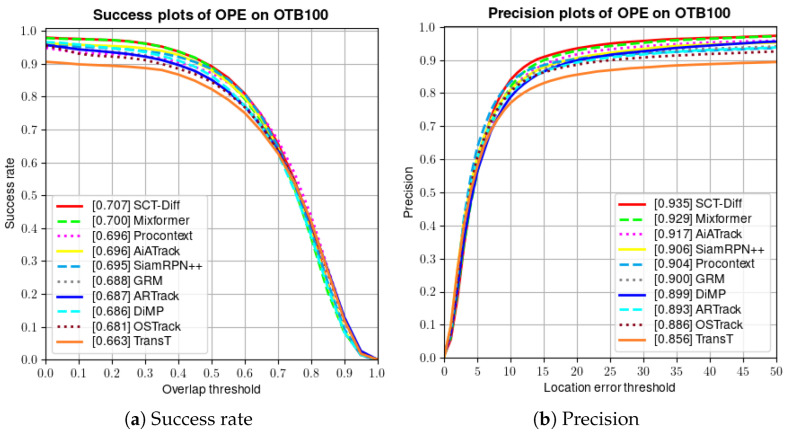
Tracking results of the proposed method on OTB-100 dataset results, (**a**) Success rate, (**b**) Precision.

**Figure 6:**
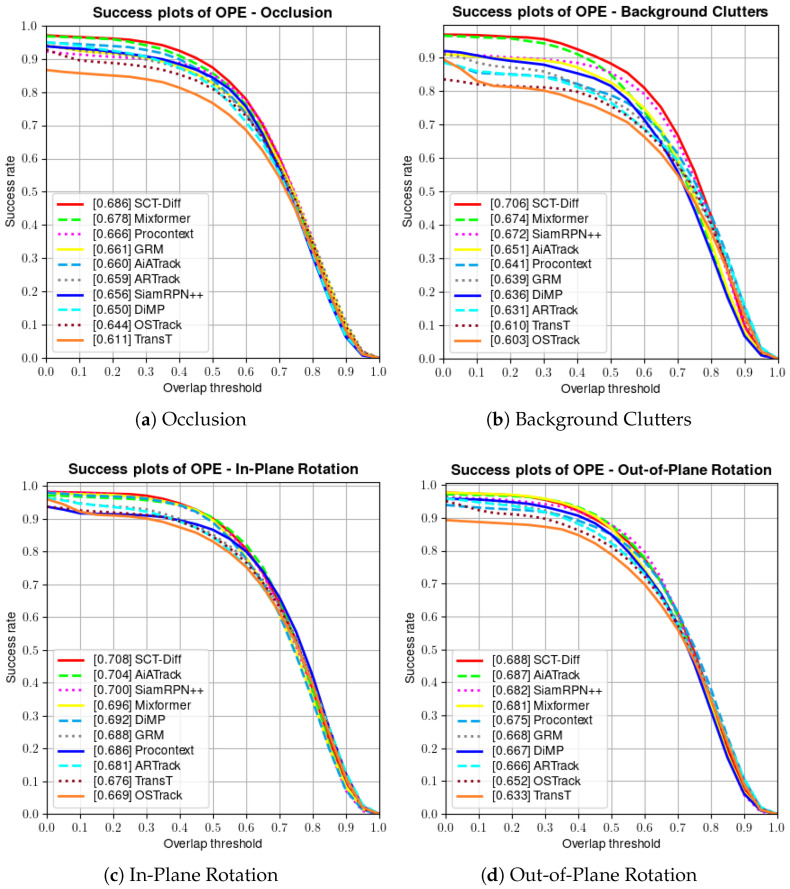
Results for the primary challenge attributes of OTB100 dataset. (**a**) Success plots of occlusion; (**b**) Success plots of background clutters; (**c**) Success plots of in-plane rotation; (**d**) Success plots of out-of-plane rotation.

For three smaller datasets, the methods involved in the comparison include MixFormer [9], ProContEXT [36], AiATrack [91], SiamRPN++ [27], GRM [28], ARTrack [15], DiMP [33], OSTrack [23], STARK [7] and TransT [4]. OTB-100 [85] is a renowned short-term tracking dataset comprising 100 videos with diverse attributes. As illustrated in Figure 5, SCT-Diff achieves success rate and precision scores of 0.707 and 0.935, respectively. Our method outperforms AiATrack by 1.1% in success rate. The latter employs multi-frame historical search regions to model target-background appearance correlations. This demonstrates the superior efficacy of leveraging video-level global spatiotemporal information. OTB-100 incorporates 11 distinct challenge attributes to evaluate tracker performance across varied scenarios. Figure 6 presents the success rate metrics of SCT-Diff’s primary results. Our approach exhibits optimal performance under Occlusion, where continuous trajectory prediction significantly mitigates target loss during transient occlusions. In the Background Clutter Challenge, SCT-Diff adapts to spatiotemporal variations by comprehensively understanding inter-background relationships. This advantage is further emphasized in the Rotation Challenge, where target transformations exhibit greater coherence.

The NFS dataset [86] is captured at a high frame rate, and our experiments utilize its 30 FPS version comprising 100 videos with significant appearance variations between consecutive frames. As shown in Table 2, SCT-Diff achieves an AUC score of 71.4%, significantly outperforming AiATrack, ARTrack, and ProContEXT that do not incorporate bidirectional temporal context. Meanwhile, the TC-128 [87] dataset is designed to assess tracker performance under complex color distributions. When integrated with the proposed diffusion-based tracking model, our method demonstrates competitive performance, as detailed in the table.

#### 4.2.6. Qualitative Analysis

**Figure 7:**
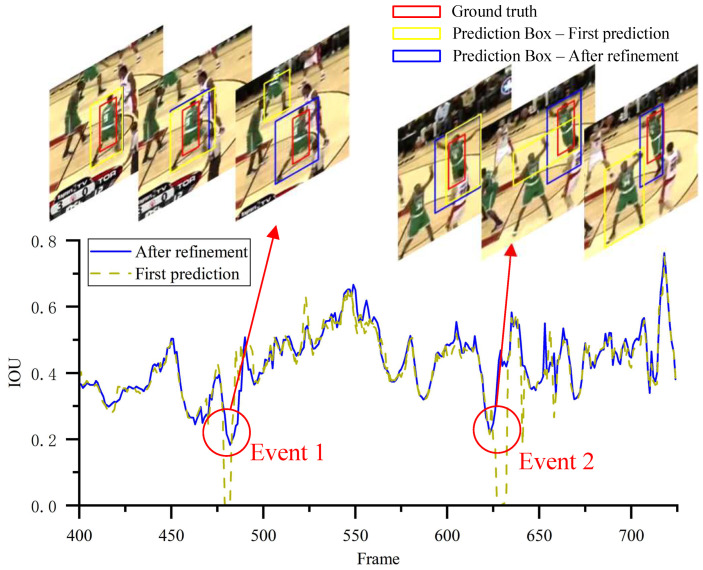
IoU before and after correction. The yellow line is the first prediction, the blue line is the corrected result, and the red line is the ground truth.

Figure 7 visualizes the tracking results of SCT-Diff on the Basketball sequence. With the diffusion framework, the target trajectory undergoes successive prediction and refinement. The visualization reveals comparable IoU performance between both trajectories relative to ground truth during smooth tracking phases. However, significant deviations emerge in the initial predictions when confronted complex tracking scenarios. At Frame #475, the primary prediction erroneously locks onto a similarly attired distractor. A comparable misidentification recurs at Frame #625. By incorporating global spatiotemporal information, our method successfully rectifies these erroneous trajectories using information from future frames. This demonstrates the framework’s effectiveness in complex scenarios requiring temporal coherence and discriminative feature analysis.

**Figure 8:**
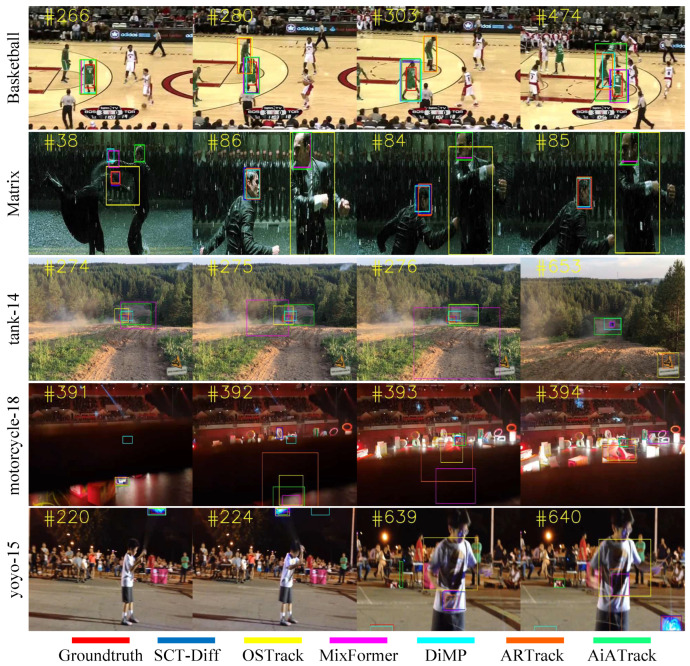
Visualization of tracking results.

To better understand our model, we present complex scenarios encountered in real-world tracking as demonstrated in Figure 8. When confronted more challenging occlusion scenarios (tank-14 #276), our method demonstrates robust prediction of reasonable target bounding boxes. Even under complete occlusion conditions (motorcycle-8 #392), SCT-Diff achieves superior tracking performance compared to ARTrack by leveraging conditioning on preceding trajectory sequences. Regarding complex backgrounds (Matrix), the occasional misassignment of bounding boxes to other instances is understandable, as humans also struggle to locate targets without visual cues. However, given prior trajectory sequences and visual information about associated targets, SCT-Diff can track occluded objects. SCT-Diff exhibits a similar ability on frame #640 of the yoyo-15 sequence. When confronted with numerous similar objects in search images (Basketball), OSTrack’s attention becomes dispersed, leading to tracking failures. In contrast, SCT-Diff maintains focus on the target by incorporating prior states. These findings substantiate our proposition that our method effectively models the sequential evolution of object trajectories in video segments.

### 4.3. Ablation and Analysis

According to the widely adopted method [17,23,76], we construct ablation studies on GOT-10k [82] for different parameters and components. The SCT-Diff settings are the same as in Section 4.1, but the number of epochs is set to 120/30.

#### 4.3.1. Video Clip Length

Video clip sets are vital for generating target trajectories. We first investigate the impact of the length of each video clip. In Table 3, the length 1 simulates a detection-based tracker. Length 4 shows significant improvement over length 1, with a noted gain of 0.9% AO. The performance of length 6 and 8 are nearly identical, with AO scores of 75.4 and 74.9, respectively. Although a length of 12 demonstrates a higher success rate than length 4 (0.3% SR_0.5_ gain), it also exhibits a decline in accurately predicting the target location than length 6 (1.4% SR_0.75_ loss). We hypothesize that this degradation results from an ineffective balance between global trajectory prediction and single-frame localization. Tracking tasks require precise localization. However, distant temporal information (such as appearance and relative position) provides limited reference value and may interfere with current localization. At the same time, processing longer video segments leads to increased computational cost and memory usage. A moderate temporal span yields the most significant improvement. Consequently, we adopt length 6 to optimize both efficiency and effectiveness.

#### 4.3.2. Time Window

**Figure 9:**
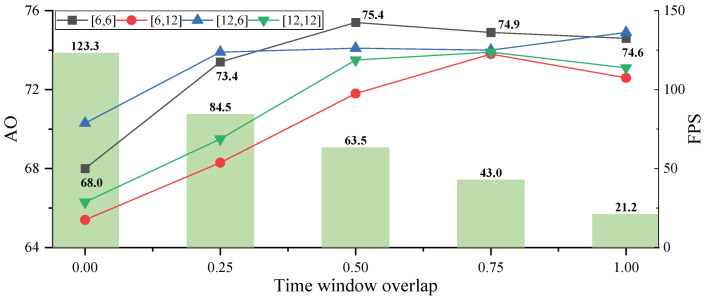
Ablation on time window.

SCT-Diff gradually predicts and corrects target trajectories by sliding a window along the temporal axis. We investigate the impact of different sliding step sizes on prediction performance across multiple training and testing trajectory lengths, as shown in Figure 9. Here, [Ntrain, Ntest] denote the trajectory lengths for the two stages, with green bins representing speed metrics ([6, 6]). As shown in Figure 9, when the overlap ratio between consecutive temporal windows is zero (i.e., no reuse of prior trajectory data for correction), the worst performance is observed (AO: 68.0%). Tracking accuracy improves as the overlap ratio increases to 0.5, where half of the trajectory is refined in subsequent predictions, achieving optimal results (AO: 75.4%). A similar trend is observed in trajectory groups of lengths 12 and 6, indicating the method’s capability to learn target motion patterns from sequential data. Further increasing the overlap ratio leads to performance saturation, while tracking speed declines significantly due to increased computational load from longer corrected trajectories. Additionally, short-term trajectory predictions yield better results than long-term forecasts. To balance accuracy and efficiency, we select [6, 6] with a 0.5 temporal window overlap ratio.

The temporal window leverages future information at two hierarchical levels to enhance tracking. At the video level (i.e., between video clips), overlapping windows harness both past frames for forward reasoning and future frames for backtracking correction. As demonstrated in Figure 9 (using the [6, 6] configuration as an example), an overlap ratio of 0 (i.e., no future information) yields the weakest performance with an AO score of 68.0. The non-zero overlap ratio and thereby incorporating future frame information markedly improves tracking accuracy, albeit with diminishing marginal returns. At the frame level, frames within a video clip are mutually visible, enabling the first frame to reference subsequent frames during inference. To isolate the contribution of this intra-segment future information, we mask the visibility of subsequent frames to previous frames within each diffusion block, thereby blocking feature interaction. This constrains the model to rely exclusively on unidirectional past information rather than bidirectional future cues. This causal variant achieves an AO score of 73.2 (Table 4), confirming that bidirectional intra-segment context also contributes meaningfully. Both levels benefit from future information, though video-level contributions remain our principal focus.

#### 4.3.3. Depth of Diffusion Layer

**Figure 10:**
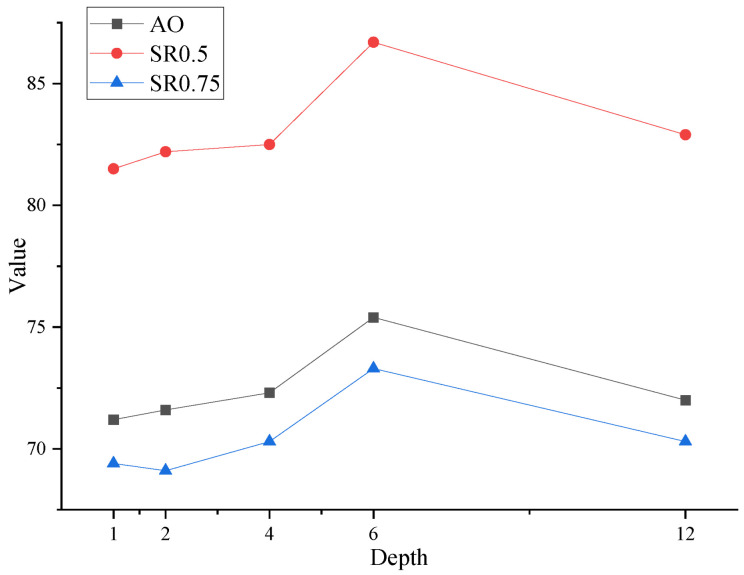
Ablation on depth of diffusion layer.

We investigate the effect of the depth of the diffusion layer in the decoder. As shown in Figure 10, appropriate decoder depth proves critical for performance. Model performance improves with increased depth within a reasonable range. Constrained by computational resources, we progressively expanded the decoder width. To achieve an optimal balance between efficiency and accuracy, we ultimately implemented a 6-layer decoder.

#### 4.3.4. Vision Expert and Language Expert

Since the encoder processes each frame independently, the decoding layer must organize frames and target states into a coherent context, namely video features and trajectory tokens. We demonstrate that continuous spatiotemporal information is effectively utilized during inference. To reduce internal dependencies within video and trajectory representations, we excised the visual experts and language experts from the decoding layer, respectively. As shown in Table 5, removing the language expert and vision expert resulted in performance declines of 3.2% and 3.7% in AO scores. This indicates that the model benefits from continuous visual and motion variations during seamless spatiotemporal tracking.

#### 4.3.5. Unified Position Reference

The proposed method operates through a unified positional reference for search window extraction across video clip frames. Selection of this positional basis critically determines the search domain near target trajectories. During training, establishing the target center position in the 0-th frame as the reference yields optimal performance. As shown in Table 6, random assignment of reference frames produced suboptimal results (72.5% success rate), while employing the mean position of all targets within the segment demonstrated the poorest efficacy, incurring a 5.3% AO reduction. The initial frame demonstrates paramount importance for reference prediction. This phenomenon aligns with the continuous motion assumption inherent to visual tracking, where search window positioning typically derives from the preceding target center. The 0-th-frame reference strategy exhibits superior compatibility with this fundamental motion continuity paradigm.

#### 4.3.6. Training Strategy

Compared to previous trackers, training SCT-Diff on video clips takes longer than on images. To establish a fair comparison, we pre-trained our model on single frames, similar to OSTrack. Two special tokens [Begin] and [End] are used to indicate the beginning and end of each frame of coordinate text, respectively. The tracking results of SCT-Diff(0) running on single frames are presented in Table 7. Subsequently, the coordinate texts are concatenated as a trajectory and fine-tuned on video clips. This two-stage training strategy improves the AO score by 1.6% and the SR_0.5_ by 3.6%, while significantly reducing the total training time.

To expand video data for training, we employed random sampling of video segments at inter-frame intervals of 1–5 frames. However, this approach induced a moderate degradation in tracking performance (1.0% in AO metrics) due to the disruption of temporal continuity.

As illustrated in Figure 3, we initially perform spatial-priority visual Mamba scanning [94]. Text features are chronologically inserted after frame features, followed by temporal frame-wise stacking. To validate different spatiotemporal inputs, we establish variants of scanning methods in Table 7. Continuous trajectory features are simply appended after video features. The model first processes video features following the spatial-priority strategy before scanning trajectory features. The optimal performance is achieved through frame-level interval insertion, potentially because frame-by-frame localization effectively reduces ambiguity in object tracking tasks. Additionally, spatiotemporal information has been effectively propagated through preceding language and vision expert modules.

#### 4.3.7. Loss Combination

The detection-based trackers perform regression in the continuous domain to predict bounding boxes, such as the combination of GIoU and L1 loss. Inspired by ARTrack [15], we convert classification coordinates into bounding boxes to facilitate similar regression predictions. As indicated in Table 8, the diffusion tracking model exhibits insensitivity to absolute error. Supervision on spatial relations achieves better results.

### 4.4. Limitations and Future Work

#### Limitations

A key limitation of the current SCT-Diff framework is its reliance on segment-based processing for object tracking. Ideally, the method would enable trajectory prediction across entire video sequences without temporal segmentation. However, extending the temporal window span imposes a significant computational burden on tracking speed, even with the two-stage training strategy. This constraint leads to two fundamental limitations: (1) it impedes the effective exploitation of longer-range temporal information, and (2) the potential positive influence of extended, coherent temporal cues on the current tracking moment remains underutilized. Future work could explore hierarchical or memory-efficient architectures to mitigate this trade-off between temporal coverage and computational efficiency.

#### Extension to Transportation Scenarios

Beyond generic object tracking, our video-level trajectories can directly feed sign interpreters (e.g., SignEye [95], SignParser [96]) as a stable visual front-end, enabling consistent sign interpretation via the natural language interface. This synergy is particularly promising for autonomous driving, where long-term sign tracking under occlusions and motion blur is critical. We plan to explore this fusion in future work, leveraging our language interface for zero-shot sign category adaptation.

## 5. Conclusions

This paper introduces SCT-Diff, a novel video-level tracking framework designed to ameliorate the limitations of conventional detection-based trackers in managing complex spatiotemporal variations. By treating VOT as vision-conditional diffusion text generation, SCT-Diff establishes seamless contextual understanding across video clips, enabling effective bidirectional utilization of temporal contexts. We bridge continuous appearance perception and motion trajectory interpretation through a Mamba-based dual-expert decoder, which integrates discrete coordinate sequence modeling with spatiotemporal video features. Moreover, the proposed seamless spatiotemporal modeling leverages future observations to progressively refine historical predictions for more coherent tracking results. Extensive experiments on mainstream benchmarks demonstrate that SCT-Diff achieves advanced performance. Extensive experiments on mainstream benchmarks demonstrate that SCT-Diff achieves state-of-the-art performance. Specifically, on GOT-10k, our method attains an AO score of 75.4% and an SR0.5 score of 86.7%, outperforming the sequence-based tracker ARTrack-1.9 in AO and surpassing the box-based diffusion tracker DffusionTrack by 1.3% in SR0.5. Additionally, SCT-Diff achieves AUC scores of 71.1% and 58.5% on the LaSOT and TNL2K datasets, respectively. In the future, we will further balance the trajectory and local search to directly reason about the whole video sequence.

## Figures and Tables

**Table 1:** Tracking results on four popular benchmarks: GOT-10k, TrackingNet, LaSOT, LaSOText and TNL2K. The first three rankings are shown in bold, underline, and italics fonts, respectively. Upward arrows denote that higher values are better for the corresponding metrics.

	Methods	GOT-10k			TrackingNet			LaSOT			TNL2K	
		AO↑	SR_0.5_ ↑	SR_0.75_ ↑	AUC↑	P_Norm_↑	P↑	AUC↑	P_Norm_↑	P↑	AUC↑	P↑
Discriminative	MDNet [90]	29.9	30.3	9.9	60.6	70.5	56.5	39.7	46.0	37.3	-	-
	ATOM [35]	55.6	63.4	40.2	70.3	77.1	64.8	51.5	57.6	50.5	40.1	39.2
	SiamRPN++ [27]	51.7	61.6	32.5	73.3	80.0	69.4	49.6	56.9	49.1	41.3	41.2
	DiMP [33]	61.1	71.7	49.2	74.0	80.1	68.7	56.9	65.0	56.7	44.7	43.4
	TrDiMP [46]	67.1	77.7	58.3	78.4	83.3	73.1	63.9	-	61.4	-	-
	TransT [4]	67.1	76.8	60.9	81.4	86.7	80.3	64.9	73.8	69.0	50.7	51.7
	STARK [7]	68.8	78.1	64.1	82.0	86.9	-	67.1	77.0	-	-	-
	AiATrack [91]	69.6	63.2	80.0	82.7	87.8	80.4	69.0	79.4	73.8	-	-
	SwinTrack-T [6]	71.3	81.9	64.5	81.1	-	78.4	67.2	-	70.8	55.9	57.1
	MixFormer-22k [9]	70.7	80.0	67.8	83.1	88.1	81.6	69.2	78.7	74.7	-	-
	OSTrack [23]	71.0	80.4	68.2	83.1	87.8	82.0	69.1	78.7	75.2	55.9	-
	GRM [28]	73.4	82.9	70.4	*84.0*	*88.7*	*83.3*	69.9	79.3	75.8	-	-
	EVPTrack [92]	73.3	83.6	70.7	-	-	-	*70.4*	80.9	77.2	-	-
Generative	ARTrack [15]	73.5	82.2	70.9	**84.2**	88.7	**83.5**	*70.4*	79.5	76.6	*57.5*	-
	SeqTrack-B [17]	*74.7*	*84.7*	*71.8*	83.3	*88.3*	82.2	69.9	79.7	76.3	54.9	-
	DiffusionTrack [76]	74.8	85.4	72.0	*83.8*	88.2	82.1	70.8	*79.8*	*76.7*	56.4	*57.3*
	MIMTrack [93]	72.6	83.2	69.3	83.1	87.7	80.9	69.1	78.8	75.7	57.9	57.7
	SCT-Diff	**75.4**	**86.7**	**73.3**	84.0	**88.8**	83.4	**71.1**	**81.0**	**77.5**	**58.5**	**58.9**

**Table 2:** Tracking results of the proposed method on NFS and TC-128 datasets.

Method	SiamRPN++ [27]	DiMP [33]	TransT [4]	STARK [7]	ProContEXT [36]	AiATrack [91]	MixFormer [9]	OSTrack [23]	GRM [28]	ARTrack [15]	SCT-Diff
NFS	50.2	61.8	65.3	65.2	70.0	67.9	65.4	64.7	65.6	63.5	71.4
TC128	57.7	61.2	59.6	60.0	58.1	58.7	60.1	54.3	54.9	55.6	63.1

**Table 3:** Ablation on length of per video clip. Upward arrows denote that higher values are better for the corresponding metrics.

Number	AO ↑	SR_0.5_↑	SR_0.75_↑
1	72.7	83.0	71.0
4	73.6	85.0	71.6
6	75.4	86.7	73.3
8	74.7	86.1	73.1
12	74.1	85.3	72.1
16	73.2	84.0	71.2

**Table 4:** Ablationon unidirectional temporal information. Upward arrows denote that higher values are better for the corresponding metrics.

Positional Reference	AO ↑	SR_0.5_↑	SR_0.75_↑
Bidirectional	75.4	86.7	73.3
Unidirectional	73.2	83.4	70.1

**Table 5:** Ablation on vision expert and language expert. Upward arrows denote that higher values are better for the corresponding metrics. Checkmarks indicate the participation of vision expert and language expert.

V.Expert	L.Expert	AO ↑	SR_0.5_↑	SR_0.75_↑
✓		72.2	82.2	70.3
	✓	71.7	82.5	69.8
✓	✓	75.4	86.7	73.3

**Table 6:** Ablation on unified position reference. Upward arrows denote that higher values are better for the corresponding metrics.

Positional Reference	AO ↑	SR_0.5_↑	SR_0.75_↑
0-th-frame reference	75.4	86.7	73.3
Random reference	72.5	83.4	70.9
Mean reference	70.1	80.3	67.6

**Table 7:** Ablation on training strategy. Upward arrows denote that higher values are better for the corresponding metrics.

Training Strategy	AO ↑	SR_0.5_↑	SR_0.75_↑
w/pre-train	75.4	86.7	73.3
w/o pre-train	73.8	83.1	71.6
Random interval	74.4	85.9	72.5
Continuous scanning	70.8	81.2	68.6

**Table 8:** Ablation on loss combination. Upward arrows denote that higher values are better. Checkmarks indicate that the corresponding loss term is included in the total loss.

CE	GIoU	L1	AO ↑	SR_0.5_↑	SR_0.75_↑
✓			72.2	81.1	66.0
✓	✓		75.4	86.7	73.3
✓	✓	✓	74.6	85.8	73.2

## Data Availability

The original contributions presented in this study are included in the article. Further inquiries can be directed to the corresponding author.
